# Enhanced audiovisual integration with aging in speech perception: a heightened McGurk effect in older adults

**DOI:** 10.3389/fpsyg.2014.00323

**Published:** 2014-04-14

**Authors:** Kaoru Sekiyama, Takahiro Soshi, Shinichi Sakamoto

**Affiliations:** ^1^Division of Cognitive Psychology, Faculty of Letters, Kumamoto UniversityKumamoto, Japan; ^2^Division of Cognitive Psychology, School of Systems Information Science, Future UniversityHakodate, Japan; ^3^Otodesigners Co., Ltd.Wako, Japan

**Keywords:** speech perception, aging, McGurk effect, response time, hearing level, lipreading, auditory-visual integration

## Abstract

Two experiments compared young and older adults in order to examine whether aging leads to a larger dependence on visual articulatory movements in auditory-visual speech perception. These experiments examined accuracy and response time in syllable identification for auditory-visual (AV) congruent and incongruent stimuli. There were also auditory-only (AO) and visual-only (VO) presentation modes. Data were analyzed only for participants with normal hearing. It was found that the older adults were more strongly influenced by visual speech than the younger ones for acoustically identical signal-to-noise ratios (SNRs) of auditory speech (Experiment 1). This was also confirmed when the SNRs of auditory speech were calibrated for the equivalent AO accuracy between the two age groups (Experiment 2). There were no aging-related differences in VO lipreading accuracy. Combined with response time data, this enhanced visual influence for the older adults was likely to be associated with an aging-related delay in auditory processing.

## Introduction

In face-to-face speech communication, perceivers use not only auditory speech information, but also visual articulatory information from the talker's face (Stork and Hennecke, 1996; Campbell et al., 1998; Massaro, 1998; Bailly et al., 2012). The use of visual information is especially prominent when auditory speech is degraded (Sumby and Pollack, 1954; Grant and Seitz, 2000; Schwartz et al., 2004; Ross et al., 2007). The contribution of visual speech to undegraded auditory speech is easily demonstrated when participants are presented with incongruent visual speech, as in the McGurk effect (McGurk and MacDonald, 1976). In the original report by McGurk and MacDonald (1976), auditory syllables with relatively high intelligibility (94% unisensory accuracy on average) were mostly perceived as auditorily wrong syllables when presented with incongruent visual speech. For example, auditory /ba/ stimuli were mostly perceived as “da” when presented with visual /ga/, indicating perceptual fusion of auditory and visual speech. Thus, the McGurk effect paradigm is a useful tool to measure the visual contribution to intelligible auditory speech.

By using this effect, it has been found that the extent of visual information use, i.e., the size of the McGurk effect varies among different populations (see Schwartz, 2010, for a review). For example, people with cochlear implants show a larger McGurk effect than people with normal hearing (Schorr et al., 2005; Rouger et al., 2008). This finding indicates that the cochlear implant users compensate for hearing impairment by heightened use of visual information. The opposite case has been found among young children in normal hearing populations. Young children show a smaller McGurk effect than adults (McGurk and MacDonald, 1976; Massaro et al., 1986; Tremblay et al., 2007; Sekiyama and Burnham, 2008). The greater reliance on auditory speech is perhaps largely due to the poorer lipreading ability of children (Massaro et al., 1986; Sekiyama and Burnham, 2008; Chen and Hazan, 2009).

The group differences in the above examples can be largely accounted for by the accuracy or confusability of unisensory information. That is, the sensory modality with less confusion plays a larger role, resulting in optimal integration as expressed by maximum-likelihood estimation or a Bayesian model (Massaro, 1987, 1998; for an improved version of the Bayesian model, see Schwartz, 2010; For a different approach, see also Braida, 1991; Grant et al., 1998). However, in some cases it is difficult to explain group differences by the unisensory accuracy alone. Language background could be one such case. For example, adult native speakers of Japanese show a smaller McGurk effect, and so a stronger auditory dependence, compared with English native speakers (Sekiyama and Tohkura, 1991, 1993; Kuhl et al., 1994; Sekiyama, 1994; Sekiyama and Burnham, 2008; also see ANOVA results of Massaro et al., 1993). Although some of these language differences may be accounted for by unisensory accuracy to some extent (Massaro et al., 1993), the Japanese-English differences in the McGurk effect could be observed when unisensory accuracy was equivalent between the two groups for both auditory and visual speech (Sekiyama and Burnham, 2008). Such a case suggests another factor affecting auditory-visual integration.

Recent neuro-cognitive studies have revealed that the integration of auditory and visual information is facilitated if the two information streams in the brain converge during an optimal time window (Van Wassenhove et al., 2005; Stekelenburg and Vroomen, 2007; Schroeder et al., 2008; also see Altieri et al., 2011, for a review). Considering the importance of such temporal characteristics, response time data can provide some insight into the integration processes in various types of populations. Sekiyama and Burnham (2008) compared unisensory response times (RTs) of Japanese and English-language children and adults, and it was found that English-language adults were faster in visual-only (VO) syllable identification (lipreading) than auditory-only (AO) identification (hearing), whereas Japanese adults' RTs were equivalent for the two conditions. Such group differences in AO-vs.-VO RT could account for language differences in the size of the McGurk effect in the auditory-visual (AV) condition. The AO − VO RT difference was not found in 6-year-olds in either of the two language groups and the McGurk effect was generally weak at this age. Based on these results, a “visual priming hypothesis” was proposed whereby the visual contribution is larger when an individual processes visual speech faster than auditory speech (Sekiyama and Burnham, 2008).

The present study investigated how aging affects auditory-visual speech perception by comparing the McGurk effect in young and older adults. In order to do this, accuracy and speed (RTs) in unisensory speech perception were both examined. Recent studies using event-related potentials (ERPs) have shown that older adults show delays in auditory processing compared with younger adults for both speech (Tremblay and Ross, 2007) and non-speech (Schroeder et al., 1995). Such neurophysiological temporal characteristics lead to an assumption that older adults have a greater visual priming effect than young adults due to delayed auditory processing. In fact, this is indirectly suggested by a previous ERP study on auditory-visual speech perception showing that multisensory temporal facilitation was greater for older adults than young adults in perceiving congruent auditory-visual spoken words (Winneke and Phillips, 2011). However, precise examinations are still necessary by measuring the RTs for each of the AO, VO, and AV conditions. Moreover, by using the McGurk effect paradigm, it is possible to investigate the relationship between temporal characteristics and how visual information is incorporated in perceived speech.

As for the susceptibility of the McGurk effect, we predicted that older adults would yield a larger McGurk effect than younger adults based on the above-mentioned delay in auditory processing. The delay may be associated with the well-documented hearing threshold decline in older adults (e.g., Glorig and Nixon, 1962; CHABA, Committee on Hearing, Bioacoustics, and Biomechanics, 1988; Pichora-Fuller and MacDonald, 2009). Combined with the fact that visual contribution is generally larger in harder hearing circumstances (Sumby and Pollack, 1954; Grant and Seitz, 2000; Schwartz et al., 2004; Ross et al., 2007), it is thought that older adults tend to utilize visual information more to compensate for their declined hearing. Such a prediction is supported by older adults' greater attention allocation to a speaker's mouth compared with younger adults (Thompson and Malloy, 2004). Also, a few studies actually suggested an aging-related increase in the McGurk effect (Thompson, 1995; Behne et al., 2007; Setti et al., 2013). However, other highly controlled studies have reported non-significant differences between young and older adults in auditory-visual speech perception (Cienkowski and Carney, 2002; Sommers et al., 2005). Cienkowski and Carney (2002) found that the aging-related difference in the size of the McGurk effect was not clear when confined to participants with age-appropriate hearing levels. They presented AV-incongruent McGurk stimuli (e.g., auditory /bi/ with visual /gi/) and older adults were compared with young controls whose auditory thresholds were shifted with noise to match the older adults. The results included some response pattern differences between groups depending on the talker and consonant, but on average, older adults integrated auditory and visual information as much as young controls. Likewise, Sommers et al. (2005) presented congruent auditory-visual speech (consonants, words, and sentences) to normal hearing older and young adults. Each participant was tested at a customized signal-to-noise ratio (SNR) to equate auditory intelligibility across individuals. The results showed the same degree of auditory-visual integration for the two groups, after factoring out lipreading performance differences. Otherwise, older adults appeared to benefit from visual speech less than younger adults.

As this statistical procedure by Sommers et al. (2005) highlights, lipreading performance was poorer in older adults compared with younger adults in the above two studies (Cienkowski and Carney, 2002; Sommers et al., 2005). This makes it complicated to compare the two age groups in terms of auditory-visual integration. The literature shows that the poorer lipreading performance of older adults depends on age and the speech material (Shoop and Binnie, 1979; Walden et al., 1993). For example, Shoop and Binnie (1979) found that aging-related decline of lipreading accuracy was observed for sentences starting from 40 years, but for consonants in a consonant + /a/ context, the decline was not very evident until 70 years. The above two studies included participants over 70 years (age range 65–74 years in Cienkowski and Carney, 2002; Mean = 70.2 and *SD* = 6.8 in Sommers et al., 2005); therefore, lowering the age limit may help reduce differences in lipreading performance between older and young groups. Consequently, this study limited older participants to the “young-old,” with an age limit up to 65 years. Also, our speech materials were consonants in a consonant + vowel /a/ context as in Shoop and Binnie (1979).

In addition, some controls were necessary over the auditory dimension to deal with age-related differences in hearing thresholds. Aging-related decline in hearing thresholds starts in the early thirties, and a significant decline occurs before the age of 65 (CHABA, Committee on Hearing, Bioacoustics, and Biomechanics, 1988). To control such age-related differences in hearing thresholds, auditory noise is often used by differing SNRs between age groups. Previous studies compared AV speech perception between older and young adults either with the same SNRs (Thompson, 1995; Behne et al., 2007; Setti et al., 2013) or calibrated SNRs between groups (Cienkowski and Carney, 2002; Sommers et al., 2005). Only in the former were age-related differences in AV performances found. Of course, the calibration of SNRs is important to investigate different groups with different hearing thresholds; however, calibrating them is not so simple. In Cienkowski and Carney (2002), the young control group received band-pass noise, which resulted in poorer AO performance in the control group compared with an older group who were not given the noise. In Sommers et al. (2005), individually customized SNRs were used for each participant, but their SNRs for 50% correct AO performance level may be too low for our McGurk effect paradigm. Considering these facts, we took two approaches. In Experiment 1, young and older adults were compared under the same auditory SNRs. In Experiment 2, the two age groups were tested under calibrated SNRs (estimated from the group results of Experiment 1 for AO perceptual equivalence). Based on the previous research, we predicted an aging-related increase in the McGurk effect in Experiment 1. If an aging-related increase is also observed in Experiment 2, it would be a novel finding.

The purpose of the present study was two-fold. First, to examine whether or not young-olds with normal hearing use visual information more than young adults. If so, the second purpose was to test the visual priming hypothesis from a previous study (Sekiyama and Burnham, 2008). The hypothesis postulates that the visual contribution will be large for those who process visual speech faster than auditory speech compared with those who process visual and auditory speech at about the same speed. We investigated whether older adults are more likely to show a greater visual priming effect in auditory-visual speech perception than younger adults. Thus, we focused on the RT differences between the AO and VO conditions as the basis for the visual priming effect. We predicted that the AO − VO RT difference would be larger for the older adults based on the delayed auditory processing reported in ERP studies (Tremblay and Ross, 2007). With the perceptually equivalent SNRs in Experiment 2, we tested whether or not older adults were still more visually influenced when unisensory auditory accuracy was the same across the two age groups. In addition, the equivalent auditory accuracy guaranteed that differences in RT represent differences in processing speed, at least for the AO condition. Before testing the hypothesis in Experiment 2 with calibrated SNRs, Experiment 1 was conducted to determine how to calibrate SNRs to obtain equivalent auditory accuracy between the younger and older adults.

## Experiment 1: speech perception performance under the physically controlled SNRs

The purposes of Experiment 1 were (1) to describe age-related differences in auditory-visual speech perception under various auditory SNRs which were physically the same for the older and younger groups, and (2) to determine SNRs for each age group under which AO accuracy was equivalent between the two age groups.

### Materials and methods

#### Participants

Thirty-four Japanese monolingual speakers participated in the experiment. The experimental protocol was approved by the Research Ethics Committee at Future University Hakodate, and all the participants filled written consent form before the experiment. Sixteen older participants (8 males, 8 females) were recruited through the City Employment Agency for Older People Hakodate. They were aged between 60 and 65 years old, and were recruited after reporting normal hearing on a self-reported basis. These people were still actively working after retirement doing part-time jobs through the Agency. Eighteen younger participants (10 males, 8 females) were university students aged between 19 and 21 years old. All of the older and younger participants had normal or corrected-to-normal vision. The experimental data were analyzed after screening the participants by hearing threshold (measured by an audiometer: Rion AA-73). Along with the criterion defined by the World Health Organization, the threshold was set to a ≤25 dB hearing level (HL) of averaged HLs of 500, 1000, 2000, and 4000 Hz. Twelve older participants met the threshold criterion (Mean ± *SD*: 18.0 ± 3.3 dB HL), while four older participants failed to meet the criterion (30.2 ± 0.8 dB HL), so were excluded from the analysis. All of the younger participants met the criterion (6.6 ± 3.4 dB HL), and were included in the analysis. The ages in the final sample were as follows: older (Mean = 62.3, *SD* = 1.8 years), younger (Mean = 20.4, *SD* = 0.9 years).

#### Stimuli

The stimuli consisted of /ba/, /da/, and /ga/ uttered by three talkers (two male and one female, native Japanese speakers). The utterances were videotaped, digitized, and edited on computer to produce AO, VO, and AV stimuli. Video digitizing was done at 29.97 frames/s in 640 × 480 pixels, and audio digitizing at 32000 Hz in 16 bit; each stimulus was created as a 2300 ms movie of a monosyllabic utterance. The duration of acoustic speech signals in each movie was approximately 290 ms on average. The movie file was edited with frame unit accuracy (33.3 ms), and the sound portion was additionally edited with 1 ms accuracy so that the sound onset was at 900 ms for each movie clip (for more details, see Sekiyama et al., 2003). Half of the AV stimuli were congruent (AVc condition: e.g., auditory /ba/ and visual /ba/, i.e., AbVb). The other half of the AV stimuli were so-called McGurk-type incongruents (AVi condition): that is, the auditory part (e.g., /ba/) is incongruent with the visual articulation (e.g., /ga/). Three kinds of McGurk-type stimuli were created by combining within-talker auditory and visual components (AbVg, AdVb, AgVb). The VO stimuli, one each for /ba/, /da/, and /ga/, were created by cutting out the audio track. In the AO stimuli, one each for /ba/, /da/, and /ga/, the video of a talking face was replaced by the still face of the talker with the mouth neutrally closed. In total, there were 9 AO stimuli (3 consonants × 3 talkers), 9 VO stimuli (3 consonants × 3 talkers), and 18 AV stimuli (3 auditory consonants × 3 talkers × 2 AV-congruent (AVc) /incongruent (AVi) types).

Auditory intelligibility was manipulated for four levels of auditory intelligibility by adding band noise (300–12000 Hz) with SNRs of 0, +6, +12, and +18 dB. The speech was always presented at 65 dB sound pressure level (SPL) and the noise level was varied. There was no noise-free condition because the previous results indicated that SNRs higher than +12 dB would result in the same performances as for a noise-free condition, at least for the younger participants (Sekiyama and Burnham, 2008).

#### Procedure

Each participant was tested individually in a sound-attenuated room. The stimuli were presented from a personal computer onto a 17-inch CRT monitor and through a loudspeaker using in-house software. Experimental conditions were blocked depending on the presentation mode (AV, AO, VO) and the SNR of the auditory stimuli (0, +6, +12, +18 dB), and there were two repetitions of each stimulus in a block (2 × 9 stimuli = 18 trials in each block in the AO and VO conditions, and 2 × 18 = 36 trials for each block in the AV conditions). Each participant was given the AV condition first. Half of the participants were tested with an AV-AO-VO order, and the other half with an AV-VO-AO order. In the AV and AO conditions, the speech was presented at 65 dB SPL at the participant's ear level, and the SNRs, 0, +6, +12, and +18 dB were determined by the intensity of the added band noise. The SNR varied across blocks in an increasing manner for half of the participants, and in a decreasing manner for the remaining participants.

Within each block, the stimuli were presented in random order. The participants were asked to watch and listen to each stimulus, decide what they perceived, and press one of three buttons for a “ba,” “da,” or “ga” response accurately and without delay. After each movie file was played, the last frame remained on the screen until one of the three buttons was pressed. Responses were made on a game controller, with input to the computer such that the responses were stored. The onset of the next stimulus was 1500 ms after the button press.

Before starting the first block of each of the AV, AO, and VO conditions, practice trials were given for nine, six, and six times, respectively, using stimuli not used in the test trials. Excluding these practice trials, the total number of trials per participant was 234 (18 trials × 4 SNRs for AO, 18 trials for VO, and 36 trials × 4 SNRs for the AV conditions). The experiment took an average of 20 min per participant for the younger group, and 30 min for the older group.

#### Statistical analysis

Statistical tests mainly focused on group-related effects; therefore, the effects of SNRs were tested only as an interaction with the age group. Analysis of variance (ANOVA) was conducted with the factors of age group (younger, older) and SNR (0, +6, +12, +18 dB) for percent correct in the AO condition, and visual influence score (AVc − AVi). An unpaired *t*-test was performed for the VO condition to examine group differences. Before each ANOVA, arcsine transformation was conducted on response accuracy to stabilize variance (*Y* = 2arcsine √*p*; *p*: proportion correct) (Howell, 1997). As a result, the visual influence scores were actually (arcAVc − arcAVi). When group-related effects were significant, planned group comparisons were always conducted for each SNR to examine in more detail the group effects. Greenhouse-Geisser correction was performed when the sphericity assumption about the variance of differences was violated, and this was reported with unmodified degrees of freedom and epsilon (ε).

### Results

**Figure 1:**
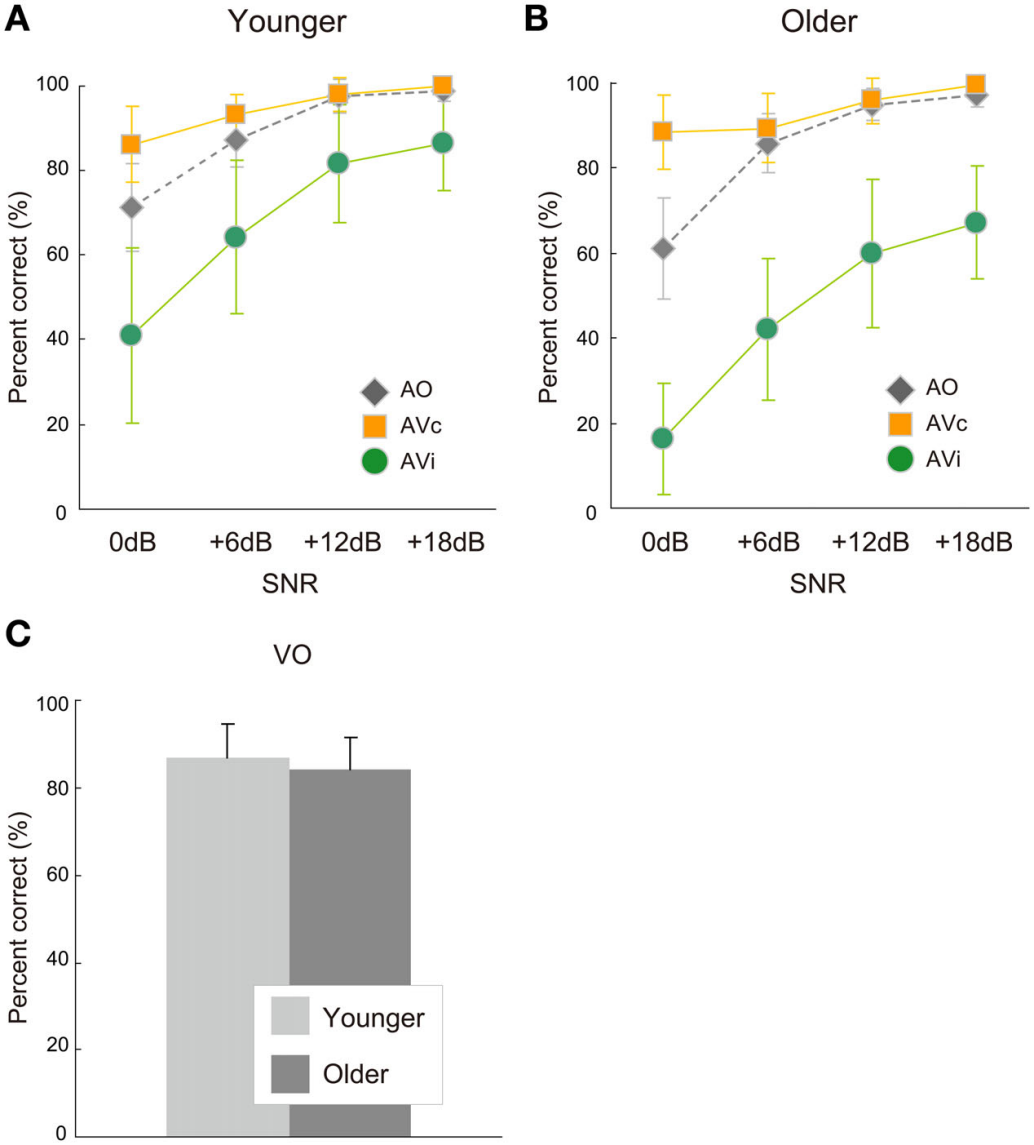
**Response accuracy of auditory-only (AO) and auditory-visual (AV) conditions in the (A) younger and (B) older groups and of (C) the visual-only (VO) condition in the younger and older groups.** Response accuracy scores are plotted for the four signal-to-noise ratios (SNRs) in the AV and AO conditions. AV conditions included two conditions: AV congruent (AVc) or incongruent (AVi) conditions.

Percent correct responses as a function of the SNR in the AO and AV conditions are shown in Figure 1A for the younger group and Figure 1B for the older group. Table 1 indicates mean response accuracy and statistical results in group comparisons. Figure 1C compares percent correct responses in the VO condition between the two groups. The correct responses were defined in terms of the auditory component of a stimulus for the AVc and AVi conditions. As described below, the older group was lower in terms of response accuracy in the AO, but not the VO condition, and yielded a larger McGurk effect in the AVi condition compared with the younger group.

**Table 1:** **Mean response accuracy (%) in Experiment 1 for younger and older groups**.

**SNR (dB)**	**Younger (*n* = 18)**					**Older (*n* = 12)**					**Group difference (*p*-value)**		
	**AO**	**AVc**	**AVi**	**AVc − AVi**	**VO**	**AO**	**AVc**	**AVi**	**AVc − AVi**	**VO**	**AO**	**AVc − AVi**	**VO**
0	71	86	41	45	87	61	88	16	72	84	0.019^*^	0.003^**^	0.223
+6	87	93	64	29		86	89	42	47		0.532	0.028^*^	
+12	98	98	81	16		95	96	60	36		0.04^*^	0.031^*^	
+18	99	100	86	14		97	100	67	32		0.122	0.002^**^	

SNR, signal-to-noise ratio; AO, auditory-only; AVc, auditory-visual congruent; AVi, auditory-visual incongruent; VO, visual-only;

* p < 0.05;

** p < 0.01.

The ANOVA for the AO condition showed a significant main effect of age group [*F*_(1, 28)_ = 11.696, *p* = 0.002, η^2^ = 0.024], while the age group × SNR interaction was not significant [*F*_(3, 84)_ = 0.919, *p* = 0.436, η^2^ = 0.005] (Figures 1A,B). Planned comparisons between groups were conducted for each SNR, and significant differences appeared at SNRs of 0 dB and +12 dB [Bonferroni: 0 dB, *p* = 0.019; +6 dB: *p* = 0.532; +12 dB: *p* = 0.04; +18 dB: *p* = 0.122]. These results indicate that the older participants were less accurate in auditory syllable identification under some (low and high) SNR conditions as compared with the younger participants.

For the VO condition, on the other hand, the older and younger groups were not significantly different [*t*_(28)_ = 1.247, *p* < 0.223, Cohen's *d* = 0.480] (Figure 1C). This indicates that lipreading performance was not different between the two age groups.

**Figure 2:**
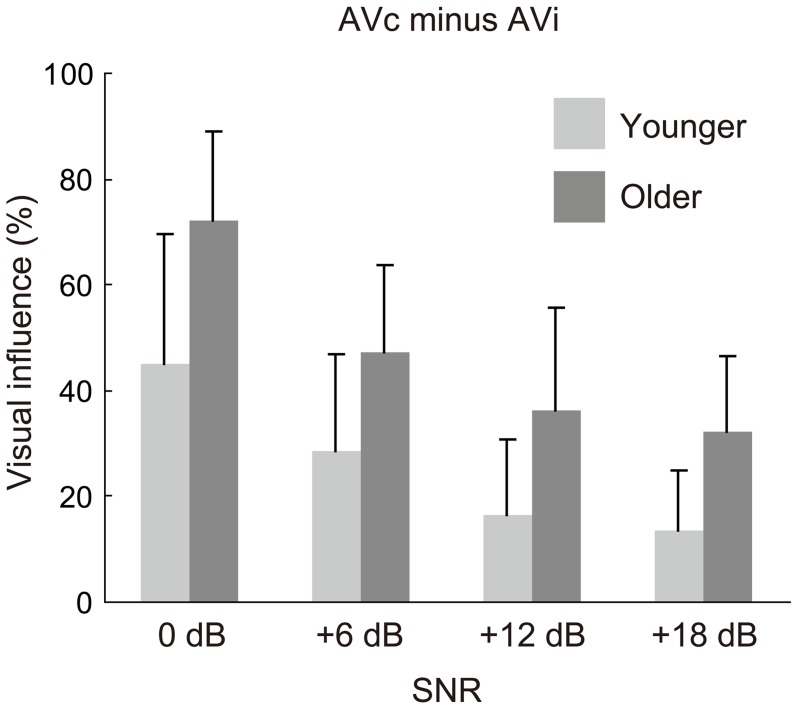
**Visual influence scores for the four signal-to-noise ratio (SNR) conditions in the younger and older groups.** The scores were calculated by subtraction of percent correct for the auditory-visual incongruent (AVi) condition from the percent correct for the AV-congruent (AVc) condition.

The visual influence scores (AVc − AVi) are shown in Figure 2. The ANOVA for this score found a significant main effect of age group, while the age group × SNR interaction was not significant [age group: *F*_(1, 28)_ = 14.164, *p* = 0.001, η^2^ = 0.188; age group × SNR: *F*_(3, 84)_ = 1.266, *p* = 0.291, η^2^ = 0.013]. Planned comparisons between groups for each SNR confirmed that the older group was more affected by visual information than the younger at each SNR (Bonferroni: all *p* < 0.04), indicating their general tendency toward greater use of visual information.

### Discussion

In Experiment 1, the AV and AO conditions were conducted for four levels of SNRs (0dB, +6dB, +12dB, +18dB), meaning that both age groups were tested under the same physical conditions. Under these experimental settings, the older group showed a larger visual influence than the younger group. For unisensory accuracy, the older group was less accurate in the AO condition than the younger group, while no significant group difference was found in VO accuracy. Taken together, the larger visual influence in the older group might be attributable to their lower AO accuracy. This is not surprising because the older group had a higher hearing threshold on average compared with the younger group, although the thresholds for both groups were within the normal hearing range. Thus, these results are basically in line with the optimal integration model.

While an aging-related increase in visual influence was observed for all four SNRs, the aging-related accuracy degradation in the AO condition was limited to high (+12 dB) and low (0 dB) SNRs. Thus, the relationship between the AO intelligibility and the visual influence was not straightforward in this experiment. To further examine the aging effect in auditory-visual speech perception, it was crucial to investigate whether the group difference in visual influence still existed when AO accuracy was equivalent between the two age groups. To do so, the results from Experiment 1 were used to determine how the AO accuracy should be equated by calibrating SNRs.

From the curves in Figures 1A,B, SNRs needed to obtain the same AO performance could be estimated: at a 90% AO accuracy, for example, the younger group's SNR was about 7 dB and the older group's about 11 dB. This was also true when we estimated each individual's SNR point for 90% AO accuracy using an interpolating method and then averaging the estimated SNRs. This group difference was used in Experiment 2 to calibrate SNRs. Perceptually-equivalent SNRs are useful to examine the visual priming hypothesis because measuring RTs should ideally be conducted under a constant accuracy (Luce, 1986).

## Experiment 2: speech perception performance under the perceptually controlled SNRs

The purposes of Experiment 2 were (1) to examine whether or not an aging-related increase in visual influence could be observed under calibrated auditory SNRs which would result in an equivalent AO accuracy for the older and younger groups, and (2) to investigate age-related changes in the visual precedence time (AO-vs.-VO in RT) to assess our visual priming hypothesis. The results of Experiment 1 revealed that mean SNRs for 90% AO accuracy were 7 dB for the younger group and 11 dB for the older group. We, therefore, set the SNRs here such that SNRs for the older group were 4 dB higher than those for the younger group. In addition, three levels of SNRs were set such that the physical SNRs cover the SNR range in Experiment 1 (0 to +18 dB) because significant group differences in the visual influence (AVc − AVi in percent correct) were observed in all SNRs in Experiment 1. As a result, Experiment 2 used following SNRs for the younger and older participants, respectively: Low (−3, +1 dB), Middle (+7, +11 dB), and High (+17, +21 dB).

### Methods

#### Participants

Fifty-one Japanese monolingual speakers participated in the experiment. The experimental protocol was approved by the Research Ethics Committee at Future University Hakodate. All of the participants completed a written consent form before the experiment. Participants were similarly recruited as in Experiment 1. Twenty-four older participants (12 males, 12 females) were aged between 60 and 65 years old. Twenty-seven younger participants (14 males, 13 females) were aged between 18 and 21 years old. All of the participants had normal or corrected-to-normal vision. Hearing tests for pure tones were conducted as in Experiment 1. The exclusion criterion for hearing threshold was the same as in Experiment 1. Seventeen older participants met the threshold criterion (16.6 ± 4.3 dB HL), while seven older participants did not (32.3 ± 4.2 dB HL) and were excluded from the analysis. All of the younger participants met the criterion (6.5 ± 3.8 dB HL). The ages of the final sample were as follows: older (Mean = 62.5, *SD* = 1.9 years), younger (Mean = 19.8, *SD* = 1.8 years).

#### Stimuli

The same speech stimuli as in Experiment 1 were used. In contrast to the same physical SNRs for the two age groups, the present experiment used perceptually equivalent SNRs for the two groups. There were three levels of SNRs (low, middle, high) for each age group. The band noise (300–12000 Hz) was always presented at 54 dB SPL and the speech level was varied so that the SNRs were +1 dB (low), +11 dB (middle), and +21 dB (high) for the older group. Similarly, the speech level was varied for the younger group so that the SNRs were −3 dB (low), +7 dB (middle), and +17 dB (high). Such SNR setting was determined based on the results of Experiment 1, indicating that older participants should be presented with speech louder by 4 dB to obtain the equivalent AO accuracy as the younger participants (see discussion of Experiment 1 and introduction of Experiment 2).

#### Procedure

The procedure was almost identical to that of Experiment 1. The only difference was that there were two kinds of VO conditions in this experiment. In addition to the VO condition with three-alternative forced choices among “ba,” “da,” and “ga” (VO3 condition), there was also a two-alternative forced choice condition (VO2 condition) in which the same three visual stimuli were presented for identification of either “ba” or “non-ba.” The VO2 condition was introduced based on a pilot experiment in which RTs for the VO3 condition often included ‘vacillating time’ between “da” and “ga” after the participants were confident that they were non-labial. We assumed that RTs for VO2 represent time for visual processing which was adequate to cause the McGurk effect (labial vs. non-labial categorization). Therefore, in terms of the visual priming hypothesis, RT differences between AO and VO2 were of our main interest. The VO2 condition was always given just before the VO3 condition. Six practice trials were given before each of the VO2 and VO3 conditions.

#### Statistical analysis

Group-related effects were mainly examined here as in Experiment 1. ANOVAs for response accuracy were conducted with factors of age group (younger, older) and SNR (low, middle, high) for auditory-related conditions (AO, visual influence calculated by arcAVc − arcAVi), and with factors of task (VO2, VO3) and age group for the VO condition. Similar ANOVAs were done for RT in the AO, AV, and VO conditions as well as for unisensory RT differences (AO − VO). For RTs, the main effect of SNR was also examined for the AO, AVc, and AVi conditions. Response accuracy was also transformed by use of the arcsine function as in Experiment 1. Raw RT data were transformed logarithmically (log_10_). When significant interaction effects were obtained, *post-hoc* analyses were performed. Planned group comparisons were always conducted for each SNR to examine the main interest of group effect. Greenhouse-Geisser correction was performed when necessary, as in Experiment 1. Lastly, correlation and partial correlation (control variable of hearing threshold) analyses were conducted between RT differences (AO − VO2) and visual influences (AVc − AVi) in all of SNRs for both groups to examine how delayed AO processing contributes to the McGurk effect size.

### Results

#### Percent correct responses

**Figure 3:**
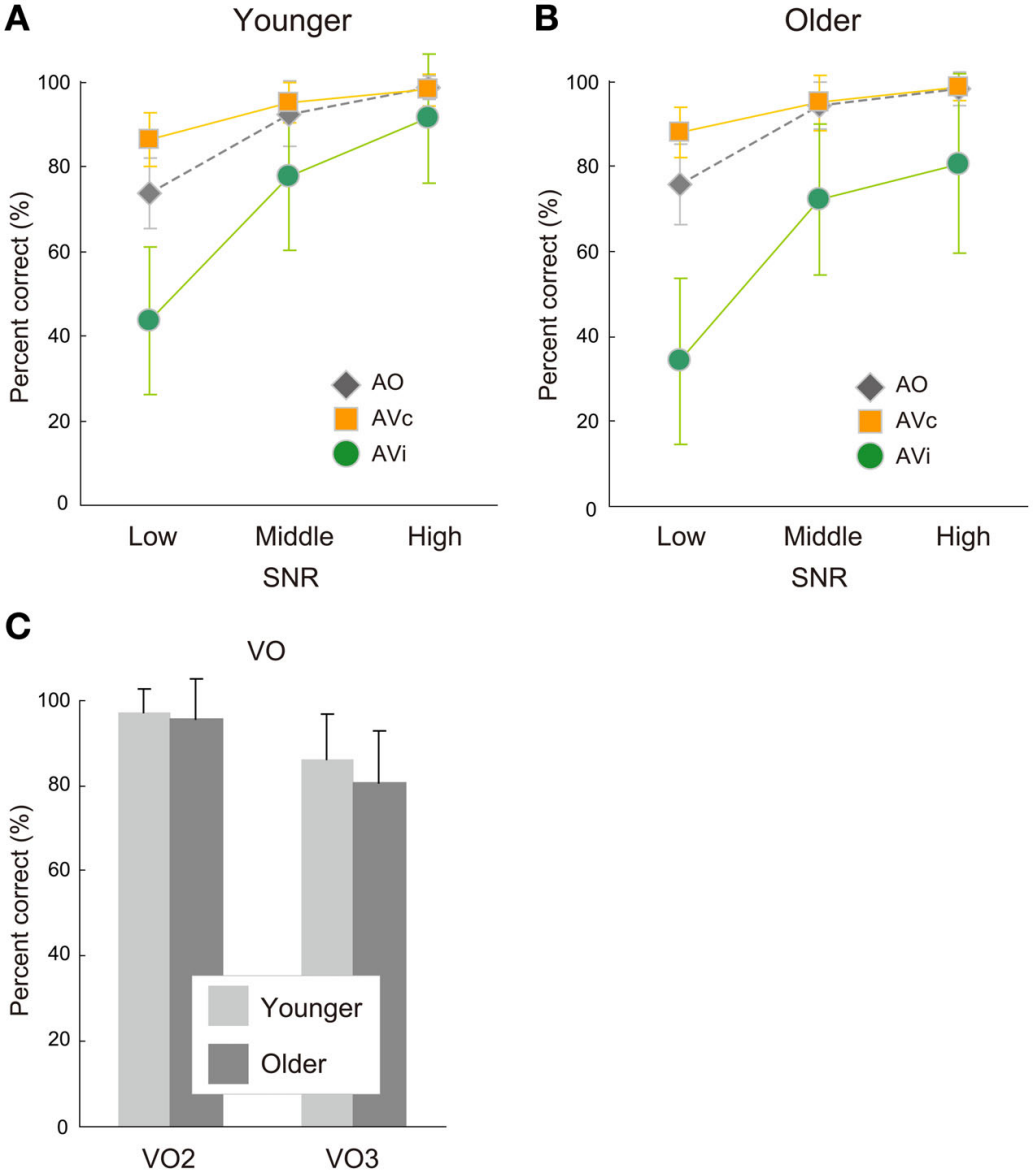
**Response accuracy of auditory-only (AO) and auditory-visual (AV) conditions in the (A) younger and (B) older groups and of (C) the visual-only condition with two-alternative (VO2) and three-alternative (VO3) choice in both groups.** Response accuracy scores in AV and AO conditions are plotted for the low, middle, and high signal-to-noise ratio (SNR) conditions. AV conditions consisted of two conditions: AV congruent (AVc) and incongruent (AVi) conditions.

Response accuracy rates for the AO and AV conditions are shown in Figure 3A for the younger group and Figure 3B for the older group. Table 2 shows mean response accuracy and statistical results in group comparisons. The response accuracy for the VO condition is also shown in Figure 3C. As described below, the older group was not significantly different from the younger in either the auditory (AO) or visual (VO) unisensory conditions, while yielding a larger visual influence in response accuracy (difference between AVc and AVi).

**Table 2:** **Mean response accuracy (%) in Experiment 2 for younger and older groups**.

**SNR**	**Younger (*n* = 27)**						**Older (*n* = 17)**						**Group difference (*p*-value)**
	**AO**	**AVc**	**AVi**	**AVc − AVi**	**VO2**	**VO3**	**AO**	**AVc**	**AVi**	**AVc − AVi**	**VO2**	**VO3**	**AVc − AVi**
Low	74	86	43	43	98	91	76	88	34	54	97	82	0.077^†^
Middle	92	95	78	17			94	95	72	23			0.321
High	99	98	91	7			98	99	81	18			0.018^*^

SNR, signal-to-noise ratio; AO, auditory-only; AVc, auditory-visual congruent; AVi, auditory-visual incongruent; VO2, visual-only two-alternative choice; VO3, visual-only three alternative choice;

† p < 0.1;

* p < 0.05.

In the AO condition, the main effect of age group or the age group × SNR interaction were not significant [age group: *F*_(1, 42)_ = 0.583, *p* = 0.449, η^2^ = 0.002; age group × SNR: *F*_(2, 84)_ = 0.284, *p* = 0.754, η^2^ = 0.001] (Figures 3A,B). This indicates that the intelligibility of the auditory stimuli became equivalent for the two groups by successfully manipulating the SNRs.

The VO performances were also similar between the two age groups (Figure 3C). Neither the main effect of age group nor the age group × task (VO2, VO3) interaction was significant [age group: *F*_(1, 42)_ = 2.013, *p* = 0.163, η^2^ = 0.001; age group × task: *F*_(1, 42)_ = 0.944, *p* = 0.337, η^2^ = 0.0003]. Thus, the two age groups did not statistically differ in terms of lipreading performance. The VO2 task was easier than the VO3 task for both groups [task: *F*_(1, 42)_ = 83.956, *p* < 0.0001, η^2^ = 0.030].

**Figure 4:**
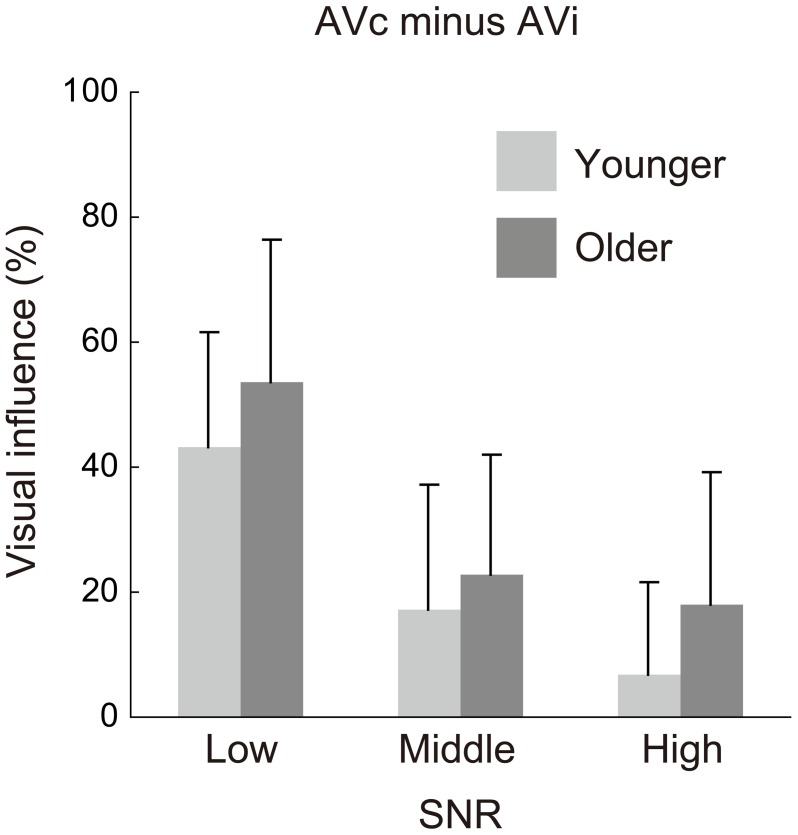
**Visual influence scores for the low, middle, and high signal-to-noise ratio (SNR) conditions in the younger and older groups. The scores were calculated by subtraction of percent correct for the auditory-visual incongruent (AVi) condition from the percent correct for the AV-congruent (AVc) condition**.

In contrast, a significant main effect of age group appeared for the visual influence score in AV speech perception [age group: *F*_(1, 42)_ = 4.990, *p* = 0.031, η^2^ = 0.054; age group × SNR: *F*_(2, 84)_ = 0.823, *p* = 0.443, η^2^ = 0.006] (Figure 4). Planned comparisons showed that the older group was more strongly affected by visual information (larger McGurk effect) than the younger, in particular, in the high SNR condition [Bonferroni: low, *p* = 0.077; middle: *p* = 0.321; high: *p* = 0.018].

#### Response time

**Figure 5:**
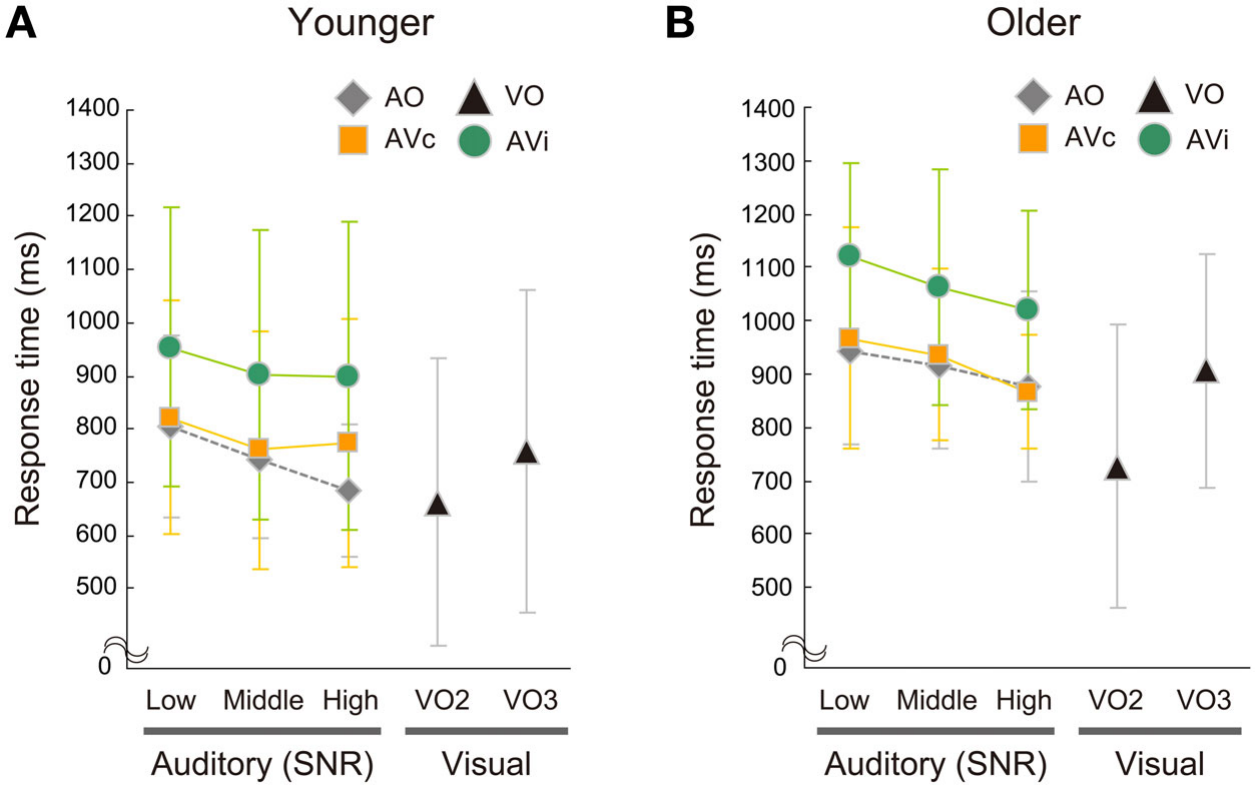
**Response times of the auditory-only (AO), auditory-visual (AV), and two-alternative (VO2) and three-alternative (VO3) visual-only conditions in the (A) younger and (B) older groups.** AV conditions included two conditions: AV congruent (AVc) and incongruent (AVi) conditions. RTs in the AO and AV conditions are plotted for the low, middle, and high signal-to-noise ratio (SNR) conditions.

Mean RTs for each condition are shown in Figure 5A for the younger group and Figure 5B for the older group. Table 3 summarizes mean RTs and statistical results in group comparisons. For both age groups, RTs were generally longer for the AVi condition compared with the AVc and AO conditions, replicating the previous results (Sekiyama and Burnham, 2008). The older group showed longer RTs in all conditions except for the VO condition compared with the younger group. Lowering the SNR in audio-related conditions generally tended to lengthen RTs.

**Table 3:** **Mean response time (ms) in Experiment 2 for younger and older groups**.

**SNR**	**Younger (*n* = 27)**							**Older (*n* = 17)**						
	**AO**	**AVc**	**AVi**	**VO2**	**VO3**	**AO − VO2**	**AO − VO3**	**AO**	**AVc**	**AVi**	**VO2**	**VO3**	**AO − VO2**	**AO − VO3**
Low	805	822	954	663	759	142	46	942	967	1121	727	906	215	36
Middle	742	761	902			79	−17	918	936	1064			191	12
High	684	773	899			21	−75	877	867	1020			150	−29
**Group difference (*p*-value)**														
**SNR**			**AO**			**AVc**		**AVi**			**AO − VO2**			**AO − VO3**
Low			0.051^†^			0.019^*^		0.014^*^			0.317			0.883
Middle			<0.0001^***^			0.003^**^		0.024^*^			0.165			0.732
High			0.010^**^			0.067^†^		0.074^†^			0.046^*^			0.522

SNR, signal-to-noise ratio; AO, auditory-only; AVc, auditory-visual congruent; AVi, auditory-visual incongruent; VO2, visual-only two-alternative choice; VO3, visual-only three alternative choice;

† p < 0.1;

* p < 0.05;

** p < 0.01;

*** p < 0.0001.

In the AO condition, ANOVA found significant main effects of age group [*F*_(1, 42)_ = 14.800, *p* = 0.0004, η^2^ = 0.216] and SNR [*F*_(2, 84)_ = 16.480; *p* < 0.0001, η^2^ = 0.044], while the age group × SNR interaction was not significant [*F*_(2, 84)_ = 1.942, *p* = 0.150, η^2^ = 0.005]. Planned group comparisons for each SNR also showed that the older group was generally slower than the younger group (Bonferroni: all of *p* < 0.02), indicating delayed auditory speech perception in older people. Higher SNR conditions tended to be faster than lower SNR conditions across groups [Bonferroni: low vs. middle, *p* = 0.051; low vs. high: *p* < 0.0001; middle vs. high: *p* = 0.010].

As in the AO condition, the older group was slower than the younger group in the AVc condition: ANOVA showed significant main effects of age group [*F*_(1, 42)_ = 7.129, *p* = 0.011, η^2^ = 0.021] and SNR [*F*_(2, 84)_ = 7.787; *p* = 0.0008, η^2^ = 0.004]. The age group × SNR interaction was not significant [*F*_(2, 84)_ = 2.103, *p* = 0.129, η^2^ = 0.001]. Planned comparisons indicated the older group was significantly or almost significantly slower than the younger group in each SNR condition [Bonferroni: low, *p* = 0.019; middle: *p* = 0.003; high: *p* = 0.067]. The low SNR condition took longer than the middle and high SNR conditions across groups [Bonferroni: low vs. middle, *p* = 0.031; low vs. high: *p* = 0.003; middle vs. high: *p* = 0.445].

In the AVi condition, the older group was also slower than the younger group. Significant main effects of age group [*F*_(1, 42)_ = 6.082, *p* = 0.018, η^2^ = 0.105] and SNR [*F*_(2, 84)_ = 5.011, *p* = 0.0124, η^2^ = 0.018] were found. The age group × SNR interaction was not significant [*F*_(2, 84)_ = 0.329, *p* = 0.721, η^2^ = 0.001, ε = 0.861]. Planned comparisons confirmed that the older group was significantly or almost significantly slower than the younger group in each SNR condition [Bonferroni: low, *p* = 0.014; middle: *p* = 0.024; high: *p* = 0.074]. The low SNR condition took longer than the high SNR condition across groups [Bonferroni: low vs. middle, *p* = 0.132; low vs. high: *p* = 0.028; middle vs. high: *p* = 0.787].

In contrast to the auditory-related conditions, RTs for the VO condition did not show significant group-related differences in either the main effect of age group or the age group × task interaction [age group: *F*_(1, 42)_ = 2.394, *p* = 0.129, η^2^ = 0.010; age group × task: *F*_(1, 42)_ = 1.805, *p* = 0.186, η^2^ = 0.001]. This suggests that the two age groups did not differ in their speed of visual syllable categorization. RT data also showed that the VO2 task was easier than VO3 task for both groups [task: *F*_(1, 42)_ = 21.484, *p* < 0.0001, η^2^ = 0.016].

**Figure 6:**
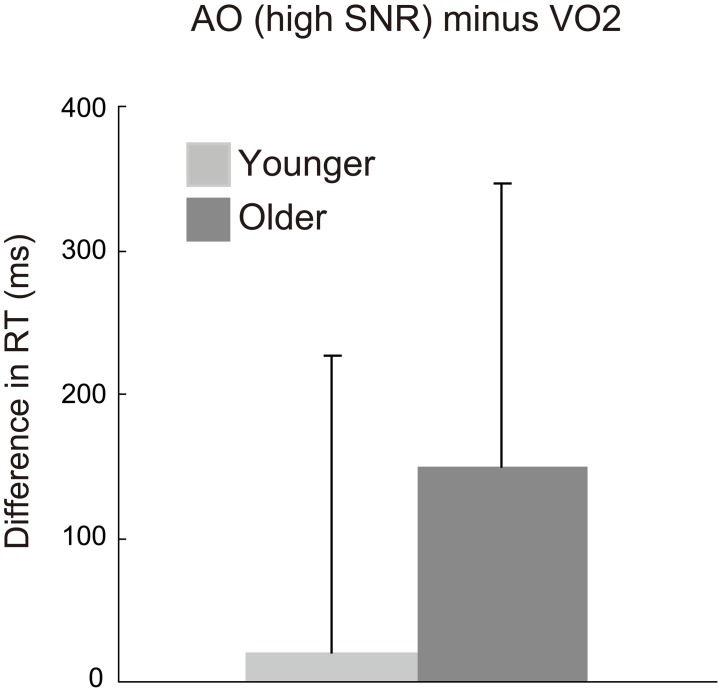
**Differences in response time (RT) between auditory-only (AO) and two-alternative visual-only (VO2) conditions in the younger and older groups. RT for the AO condition is from the high signal-to-noise (SNR) condition**.

The differences in RTs between the AO and VO conditions were compared between age groups. Although the main effect of group and the interaction effect of group × SNR were not significant in the ANOVA, the group comparison for the high SNR condition was especially of our interest, because the group difference in the McGurk effect was significantly observed in the high SNR condition in response accuracy. The planned unpaired *t*-test showed that the temporal difference was larger in the older group than the younger group for the AO relative to VO2 condition in the high SNR condition [*t*_(42)_ = 2.055, *p* = 0.046, Cohen's *d* = 0.65] (Figure 6). Such group differences did not reach significance in the middle and low SNR conditions [middle: *t*_(42)_ = 1.415, *p* = 0.165, Cohen's *d* = 0.45; low: *t*_(42)_ = 1.012, *p* = 0.317, Cohen's *d* = 0.32]. There were no significant group differences in AO − VO3 [high: *t*_(42)_ = 0.645, *p* = 0.522, Cohen's *d* = 0.20; middle: *t*_(42)_ = 0.344, *p* = 0.732, Cohen's *d* = 0.11; low: *t*_(42)_ = 0.149, *p* = 0.883, Cohen's *d* = 0.05].

**Figure 7:**
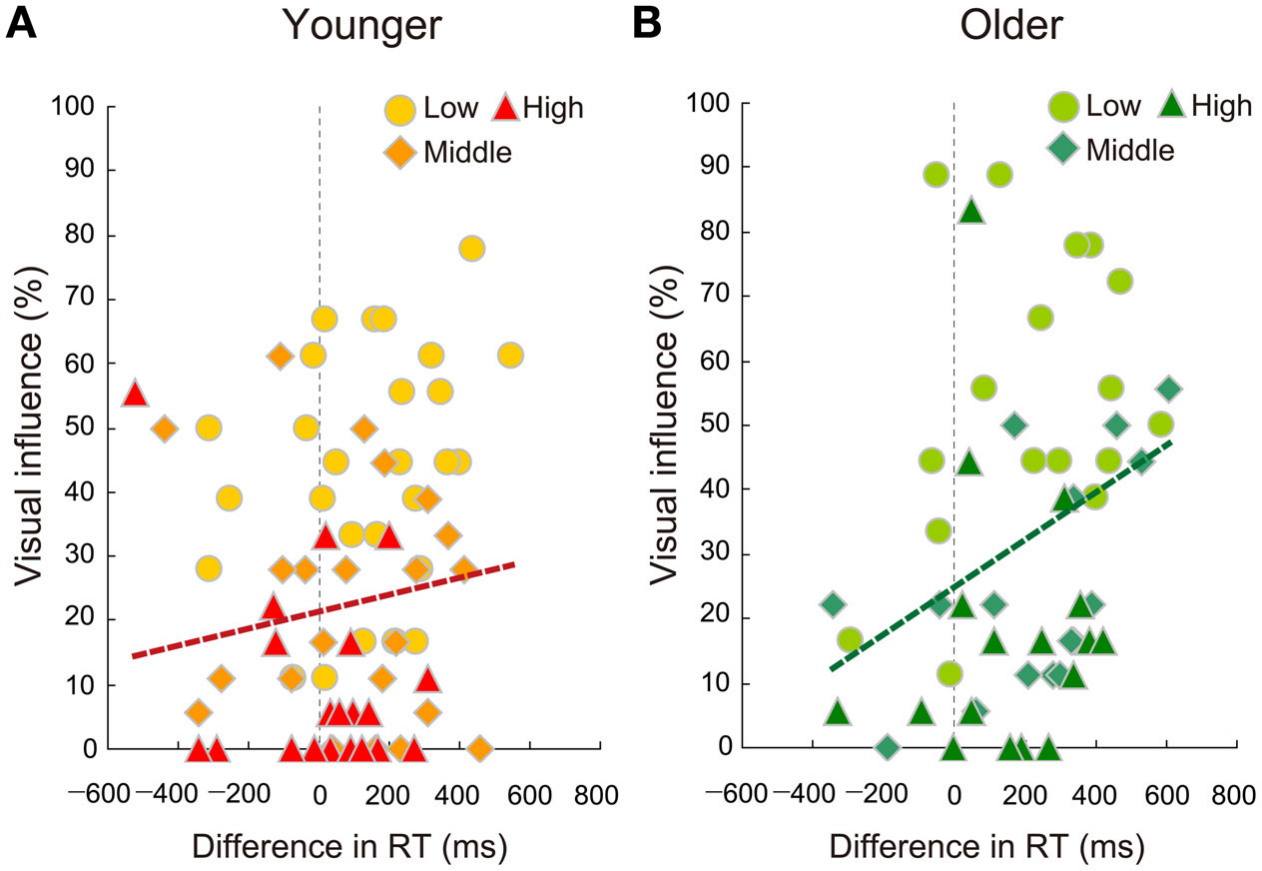
**Relationships between difference in RT (auditory-only minus two-alternative visual-only conditions) and visual influence in percent correct (auditory-visual congruent minus incongruent conditions) using all data from the three signal-to-noise ratios (Low, Middle, High) in the (A) younger and (B) older groups.** The older group, but not the younger group, showed a significant correlation.

Finally, continuous correlation analyses were conducted between RT differences (AO − VO2) and percent visual influence scores (AVc − AVi) for both the younger and older groups, using all of three SNR data. Although the younger group did not show a significant correlation [*r* = 0.127, *p* = 0.258; *n* = 81] (Figure 7A), the older group yielded a significant positive correlation [*r* = 0.337, *p* = 0.016; *n* = 51] (Figure 7B). Such significant correlation relationship for the older group remained significant under the control of hearing thresholds [older (*n* = 48): ρ_*XY·Z*_ = 0.374, *p* = 0.008; younger (*n* = 78): ρ_*XY·Z*_ = 0.128, *p* = 0.259]. As inferred from this, there was no significant correlation between the RT differences (AO − VO2) and hearing thresholds in the older group (*r* = −0.094, *p* = 0.512). These results indicate that more delayed AO perception (being positive in difference in RT) was related with larger McGurk effects in the older group.

In summary, in discrete analyses, the AO − VO2 RT difference was significantly larger for the older group in the high SNR condition, and this coincided with the result for the visual influence score for which planned comparisons showed a significant age difference in the high SNR condition. In continuous analyses across all of the SNR conditions, the older group showed a significant correlation between the size of the McGurk effect and the unisensory RT difference (AO − VO2), indicating that the larger McGurk effect is associated with more delayed AO perception. These results support the visual priming hypothesis.

### Discussion

Experiment 2 compared older and younger participants not only in terms of response accuracy, but also RT; therefore, we calibrated the SNR of auditory stimuli so that the auditory intelligibility was equivalent for both age groups. With a difference of 4 dB of SNR between the two age groups, the older and the younger were tested in low (1 or −3 dB), middle (11 or 7 dB), and high (21 or 17 dB) SNRs in the AV and AO conditions. The results showed that the McGurk effect was still stronger for older than for younger adults under equivalent auditory intelligibility. The two age groups also showed equivalent accuracy of VO performance. Because the two age groups were equivalently accurate in both AO and VO performances, the age difference in the McGurk effect needs to be explained by a factor other than unisensory accuracy.

Response times revealed that the delay due to aging was large in conditions that included auditory stimuli (AO, AVi, and AVc), whereas there was no such delay in lipreading; this was especially so for labial–non-labial categorization (VO2). Because we assumed that RTs for VO2 represent time for visual processing which was adequate to cause the McGurk effect (labial–non-labial categorization), we focused on the visual precedence time in the binary lipreading condition (AO − VO2 in RT). The visual precedence time (AO − VO2) was significantly larger for the older group than the younger group in the high SNR condition, but not in the middle and low SNR conditions. This was in accordance with the fact that the aging-related increase in the visual influence on accuracy tended to be more pronounced for the high SNR condition. These results suggest that the older participants' larger visual precedence due to delayed auditory processing (particularly in the high SNR condition) is related to a larger visual influence. The co-occurrence of the larger visual precedence and the larger visual influence in the older group is consistent with our visual priming hypothesis.

Moreover, the within-group correlation analysis across all SNRs found a significant correlation between the size of the McGurk effect and the unisensory RT difference (AO − VO2) in the older adults: The larger McGurk effect was associated with the larger visual precedence, supporting the visual priming hypothesis. Such an association was not found in the younger participants, thus the association in the older participants seems to be based on the aging-related auditory delay.

On the other hand, the visual precedence time for three-alternative lipreading conditions (AO − VO3 in RT) was not significantly different between the two age groups. This may be a general tendency of the elderly who attach importance to accuracy rather than speed when the task is difficult (in VO3).

## General discussion

This study investigated whether or not older adults with normal hearing and preserved lipreading use more visual speech information than younger adults in auditory-visual speech perception. Particularly, we intended to examine our visual priming hypothesis that emphasizes the amount of temporal precedence of VO speech processing relative to AO processing as a cause of the aging-related increase in visual influence.

Previous studies on aging-related differences in auditory-visual speech perception presented auditory stimuli to older and younger adults either under the same SNRs (Thompson, 1995; Behne et al., 2007; Setti et al., 2013) or calibrated SNRs (Cienkowski and Carney, 2002; Sommers et al., 2005), and only the same-SNR settings found significant aging-related differences. Among the above studies, only some studies conducted screening of the participants based on hearing thresholds (Cienkowski and Carney, 2002; Sommers et al., 2005; Setti et al., 2013). Concerning the age range of the participants, control was not so strict in most of these studies. In fact, studies including older adults over 70 years have often revealed poorer lipreading in older adults, which would make it complicated to assess aging-related changes in AV integration. Our strategies were (1) to use both the same SNRs and calibrated SNRs, (2) to exclude participants with clinically declined hearing, and (3) to minimize the aging-related decline in lipreading by setting an age range of older adults between 60 and 65 years.

We found that the visual influence was greater in the older adults compared with the young adults not only in the same SNRs, but also in the calibrated SNRs. Based on the effect size of the main effect of age group (η^2^ = 0.188 in Experiment 1; η^2^ = 0.105 in Experiment 2), the aging-related difference in the visual influence were larger under the same SNRs than the calibrated SNRs. This is reasonable because the same-SNR setting did not correct the aging-related poorer AO performance for the older adults, so it would have led to a greater visual influence on them as predicted from optimal integration models (Massaro, 1987, 1998; Braida, 1991; Grant et al., 1998; Schwartz, 2010).

The novel finding of the present study is that the aging-related increase in the visual influence was significant even under the calibrated SNRs. Importantly, the calibration was successful as confirmed by non-significant age group differences for unisensory AO accuracy. Therefore, for the first time, the aging-related increase in visual influence was revealed after controlling for the hearing decline of older adults. In the accuracy data in Experiment 2, there were no age group differences in unisensory performance not only in the AO, but also in the VO conditions. Nevertheless, the multisensory AV integration differed between the two age groups. Therefore, the differential AV integration between the two age groups must be attributable to some factors other than unisensory accuracy: this is a starting point to examine our visual priming hypothesis, which was supported by the present RT results.

The RT difference between older and younger adults was constant in audio-related conditions (AO and AV), while no such delay in RTs for older adults relative to younger adults was observed in the VO condition. Of importance, this aging-related auditory delay could be persistent when the visual labial–non-labial decision (VO2) was not delayed. Thus, the older group's larger RTs in the AO condition were not attributable to general response slowing, but to the modality-specific delay in auditory processing. Consequently, the visual precedence time (AO − VO2) was significantly longer in the older than the younger adults in the high SNR condition. In accordance with this, the aging-related increase in visual influence tended to be more pronounced in the high SNR condition, yielding a larger McGurk effect in the older adults. Moreover, the correlation analyses within the older group across SNRs indicated that more delayed RT is associated with the larger McGurk effect. Therefore, the visual priming hypothesis was supported in two aspects: One is the group differences in the high SNR condition, and the other is the correlation within the older group.

The delayed auditory processing of older adults has also been found in studies using ERPs both for speech (Tremblay and Ross, 2007) and non-speech (Schroeder et al., 1995). Furthermore, ERPs for AV congruent stimuli revealed that the temporal facilitation of speech processing by visual speech is greater for normal hearing older adults compared with younger adults (Winneke and Phillips, 2011). Such a temporal, visual facilitation is thought to be due to anticipation provided by visual lipread information that starts a few hundred milliseconds earlier than the onset of auditory energy in natural speech articulation (Van Wassenhove et al., 2005; Stekelenburg and Vroomen, 2007; Arnal et al., 2009). The temporal facilitation in ERPs was also observed for non-speech events where the anticipatory visual motion precedes the sound, for example, in hand clapping, but not in events where visual motion and sound start at the same time, for example, paper tearing (Stekelenburg and Vroomen, 2007). Thus, anticipatory visual motion may predict when a sound will occur (Stekelenburg and Vroomen, 2007) and what phonemes are candidates (Van Wassenhove et al., 2005). The visual precedence time in RT in the present older adults could be a measure of temporal information about how much in advance the visual anticipation is generated relative to the auditory perception. The present results suggest that visual anticipation may function well to influence auditory processing, when visual precedence time is at least about 100 ms, as observed in the RT difference between AO and VO2 in the older adults.

Concerning SNRs and aging-related performance differences, the relationship between AO accuracy and the visual influence (AVc − AVi) was not always simple. In experiment 1, significant group differences in AO accuracy were found in two SNRs, while group differences in the visual influence score were significant at all SNR levels. This seems in accordance with the fact that the effect of lipreading on AV accuracy is not additive to AO accuracy, but in a multiplicative way (e.g., Braida, 1991), thus, small or non-significant differences in AO conditions could turn into large differences in AV conditions (Sumby and Pollack, 1954). In Experiment 2, we used calibrated SNRs to eliminate group differences in AO accuracy, thus it is naturally expected that group differences in the visual influence would be observed in more limited way compared with Experiment 1. In fact, a significant group difference in visual influence score was found only at the high SNR.

It was unexpected that the group difference was more prominent at the high SNR than the middle and low SNRs. Why was this? It may have been due to the relativity in RTs between the AO and VO conditions. Although the older group showed a constant AO delay relative to the younger group at each SNR, the RTs became longer as the SNR became lower for both groups. As a result, the visual precedence time (AO − VO2), which was almost zero for the younger adults at the high SNR, reached a substantial amount at the middle and low SNRs for the younger, as well as for the older, adults (Figure 5). This caused a substantial degree of visual influence on both groups in the middle and low SNRs (Figure 4), which may have resulted in reduced age group differences.

On the other hand, there may be a case in which the visual priming hypothesis does not hold. In the context of non-speech processing, a previous study demonstrated that multisensory facilitation on RT of simple detection relative to unisensory detection was greater for older adults than young adults (Peiffer et al., 2007). They used lights and white noise as stimuli, and a multisensory condition was presented to them at the same time. An aging-related increase in multisensory facilitation was still found even when unisensory detection was equally fast for both age groups. The time course in which visual and auditory streams are integrated may be different depending on stimuli (dynamic visual motion vs. static light, and anticipatory vs. abrupt visual cues) and task (categorization vs. detection).

Recently, individual differences in the McGurk effect among young perceivers were studied in terms of the “temporal binding window” (Stevenson et al., 2012). These authors found that persons who are more sensitive to beep-flash asynchrony (thus with smaller temporal binding window) are more susceptible to the McGurk effect. This suggests that mechanisms for detecting auditory-visual simultaneity are also relevant to some extent for integration of auditory and visual speech information. Could older adults with delayed auditory processing have any drawbacks to auditory-visual simultaneity detection? One possibility is that the delay of auditory relative to visual processing may be perceptually canceled as the older adults adapt to the aging-related delay and recalibration takes place as found for experimental lags in young adults (Fujisaki et al., 2004). If so, the temporal binding window itself may not be a source of aging-related differences in the McGurk effect. However, the extent to which the temporal binding window accounts for individual differences in the McGurk effect may differ between age groups. In the present study, the visual precedence (that is, auditory delay) was associated with the size of the McGurk effect only in the older adults. Therefore, the young adults' individual differences in the McGurk effect should be accounted for by the other factors, such as the temporal binding window, whereas those of the older adults are possibly accounted for by both the auditory delay and temporal binding window.

Finally, we should mention the inconsistency between the present findings of a larger McGurk effect in the older group and the previous findings (Cienkowski and Carney, 2002; Sommers et al., 2005). A few factors may have contributed to the inconsistency. One is the age range of the participants: we excluded those over 66 years to minimize lipreading decline (Shoop and Binnie, 1979). Another critical difference may be the range of SNRs: we used a wider range of SNRs including much milder SNRs compared with the previous studies. These factors may have partially contributed to the inconsistency between the present and previous studies.

In conclusion, this study demonstrated that native Japanese speaking older adults used more visual speech information than their younger counterparts, and were more susceptible to the McGurk effect when tested with stimuli containing equivalently intelligible auditory speech. From the RT data, the enhanced visual influence on the older adults was likely associated with an aging-related delay in auditory processing. The delay was observed despite the equalized AO accuracy between the two age groups, presumably representing aging-related changes in higher order neural processes that are hard to observe by hearing thresholds alone (Pichora-Fuller and MacDonald, 2009). Time-related measures such as RTs and ERPs are important to assess older adults' auditory perception. In this study, there was no correlation between hearing thresholds and delay in auditory RT, indicating that the two factors are dissociable. Thus, among the older adults with normal hearing, it may be that the delay in cortical auditory processing, rather than peripheral sensory sensitivity, is more critical for the greater visual influence. Furthermore, it was previously shown that the RT difference between auditory and visual speech perception was larger for young native English speakers than for young Japanese speakers (Sekiyama and Burnham, 2008). It will be of interest to clarify in the future whether or not the procedure used in the present study can reveal an aging-related increase in visual precedence in English speaking populations as in Japanese.

### Conflict of interest statement

The authors declare that the research was conducted in the absence of any commercial or financial relationships that could be construed as a potential conflict of interest.
